# An overview of the “Protecting Cantonese Movement” in Guangzhou (2010–2021)

**DOI:** 10.1186/s40862-022-00165-2

**Published:** 2022-09-15

**Authors:** Yaling Li, Yeqin Kang, Dan Ding, Nianqing Zhang

**Affiliations:** 1 South China Normal University, Guangzhou, China; 2 School of English Education, Guangdong University of Foreign Studies, Xiaoguwei, Panyu District, Guangzhou, 510006 China

**Keywords:** The “Protecting Cantonese Movement” (PCM), Language maintenance, Sociocultural context, Sociolinguistic context, Media environment, Guangzhou

## Abstract

A decade ago, an online survey resulted in the “Protecting Cantonese Movement” (PCM) in Guangzhou, Guangdong Province, China, around which some articles were produced. Based on the bibliometric analysis of data retrieved from the *China National Knowledge Infrastructure (CNKI)*, the *Web of Science*, and *Google* Scholar keyword searches during 2010 and 2021, this paper reviews the PCM literature to summarize its major features and general trends. It reveals that the published journals or newspaper articles focus on interpretating PCM, analyzing its causes from sociocultural, sociolinguistic, socioeconomic and media environmental perspectives, and proposing countermeasures and suggestions at political, ideological and sociolinguistic environmental levels. It concludes that PCM is of far-reaching significance to the continuous research ranging from Cantonese preserving, promotion of Mandarin, language planning, language policy, and language conflict to language ecology.

## Introduction

Language, as a resource (Ruiz, 1984), will be depleted without good protection, development and planning (Chen, 2008; Xu, 2008, Ester, Tuba & Nidza, 2019). Language maintenance has been a hot issue since the late 20th century (Guardado, 2002; Garcia, 2003; Fishman, 2004; Decapua et al., 2009; Gao, 2012). It is hardly surprising that with the growth of a dominant language (e.g., English) around the world, some languages will face the danger of demise in the future (Crystal, 2000). Accordingly, the use of a local language in the public domain is one of the key drivers for its maintenance. In China, with the promotion of Mandarin, Cantonese maintenance has also drawn much attention from researchers, especially due to the “Protecting Cantonese Movement (PCM)” initiated in July 2010 (Chen, 2011; Zeng, 2010; Zhan, 2011; Qu, 2011a, 2011b; Li, 2012; Gao, 2012, 2015, 2017; Li et al., 2019). The PCM was reportedly caused by a document submitted by the Guangzhou Committee of the Chinese People’s Political Consultative Conference (CCPPCC) to the municipal government proposing the switch from the use of Cantonese to Mandarin on local television channels to attract visitors worldwide in the Asian Games 2010 (Gao, 2012; Wang & Liu, 2013, 2013; Xiao, 2010). In this regard, Guangzhou CCPPCC conducted an online survey entitled “Questionnaire on Guangzhou TV Station Broadcasting”. One of the questions was “Which language is better for the Guangzhou News Channel to broadcast in, Mandarin or Cantonese?” Unintentionally, this survey question turned into an unprecedented online debate about “promoting Putonghua (Mandarin)” vs. “defending Cantonese”, resulting in a thousand-person gathering. Between July 25 and August 1, 2010, some people in Guangzhou held rallies and marches under the slogan of “Defending Cantonese”, expressing their support for Cantonese with practical actions (Wang & Liu, 2013, 2013).

Triggered by PCM in 2010, researchers have published articles on the maintenance of Cantonese over the last decade. Based on these publications, this paper overviews researchers’ concerns and opinions on PCM and language maintenance. Altogether, three questions are addressed: (1) What are the researchers’ interpretations of PCM? (2) What are the causes of PCM in their view? (3) What countermeasures have they proposed for language maintenance?

## Data collection

The paper uses bibliometric analysis to cluster the literature to be reviewed. Although a large number of articles were searched under the keyword Cantonese, a limited number were identified as “Protecting Cantonese Movement” oriented.

### Data sources

Literature in Chinese and English was collected.

Literature in the Chinese language was retrieved from the China National Knowledge Infrastructure (CNKI) database. As the PCM occurred in 2010, the time span was set as 2010–2021. The Chinese literature search keywords were “protecting Cantonese”, “promoting Mandarin in Canton”, “controversy between Mandarin and Cantonese” or “Guangdong dialect”, with “full text” as the search field. Through quick browsing of the abstracts (reading the full text if necessary), the authors retained literature with “protecting Cantonese” and “Cantonese” as the research objects or main cases. Ultimately, 55 journal articles and 45 newspaper articles were screened out.

English literature was retrieved through the “Web of Science” database and “Google Scholar”, with “Guangzhou Cantonese”, “Cantonese Conflict”, “Protecting Cantonese Movement” or “Guangzhou Dialect” as the English searched keywords during 2010–2021. A total of 52 articles were retrieved and further refined through manual reading. Finally, 10 valid studies were obtained.

### Time distribution of literature

**Fig. 1:**
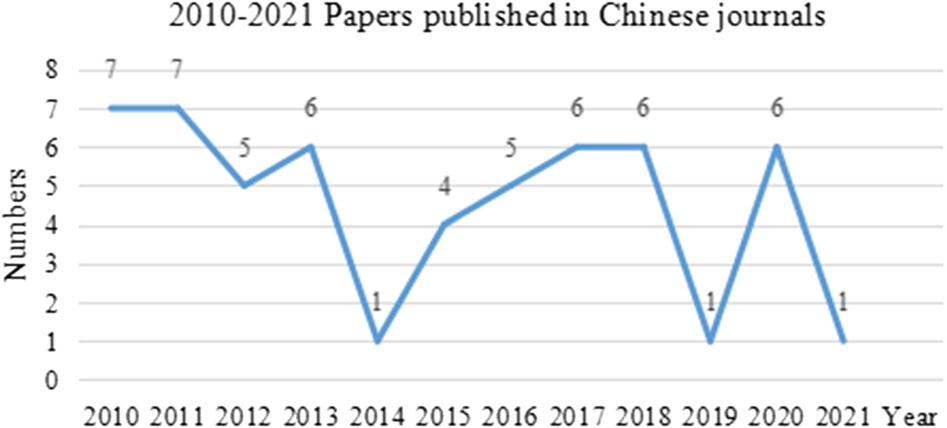
2010–2021 Papers published in Chinese journals

**Fig. 2:**
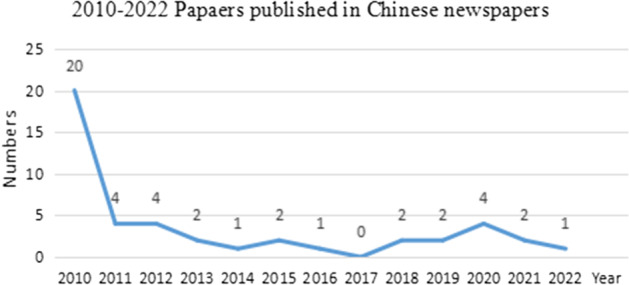
2010–2021 Papers published in Chinese newspapers

**Fig. 3:**
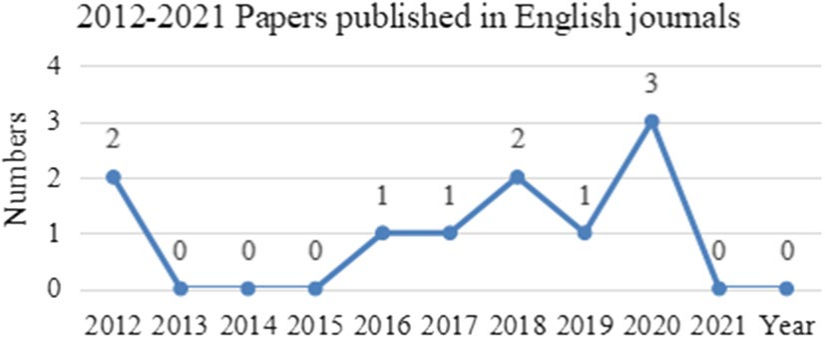
2012–2021 Papers published in English journals

To better understand the entire process of PCM from a macro perspective, this paper conducts a preliminary statistical analysis of the Chinese literature, and the results are shown in Figs. 1, 2 and 3.

Judging from the year of publication during 2010–2021, the number of published articles in Chinese journals waves, showing that this topic continuously attracts research attention (Fig. 1). Most scholars made comments on PCM within two or three years after 2010, but few performed continuous research except Qu Shaobing (2011a, 2011b, 2012, 2016, 2020, 2021). Some studies discuss the incident of “Protecting Cantonese”, but the themes are scattered.

The number of newspaper articles on preserving Cantonese decreased sharply (Fig. 2) after 2011, which also reflected that people’s concerns about the “battle of Cantonese or Mandarin” diminished over time or became more rational.

The 10 English articles were published during 2012–2020 (Fig. 3), 3 of which were written by Xuesong Gao (2012, 2015, 2017), and the most frequently cited article (32 times) was published by Gao in 2012, indicating less attention to this research field.

### Distribution of literature types

Statistics showed that the 55 Chinese articles were published unevenly in 44 different journals. In terms of their attributes, these journals covered a variety of disciplines: sociology, philosophy, linguistics, culture, etc. Some journals were edited by universities, including comprehensive edition and social science edition. The majority of 45 newspaper articles, however, was published in mainstream media such as *Southern Daily*, *Guangzhou Daily*, *People’s Daily*, China Education News, China Culture News, etc. The 10 English articles were distributed in periodicals and conferences proceedings, with two on *Journal of Multilingual and Multicultural Development*, which indicated the worldwide attention to PCM, and a wide range of thinking in the field of multilanguage and multiculture. The PCM aroused great repercussions in Cantonese-speaking Guangzhou, China nationwide, and worldwide.

### Topic distribution of literature

**Fig. 4:**
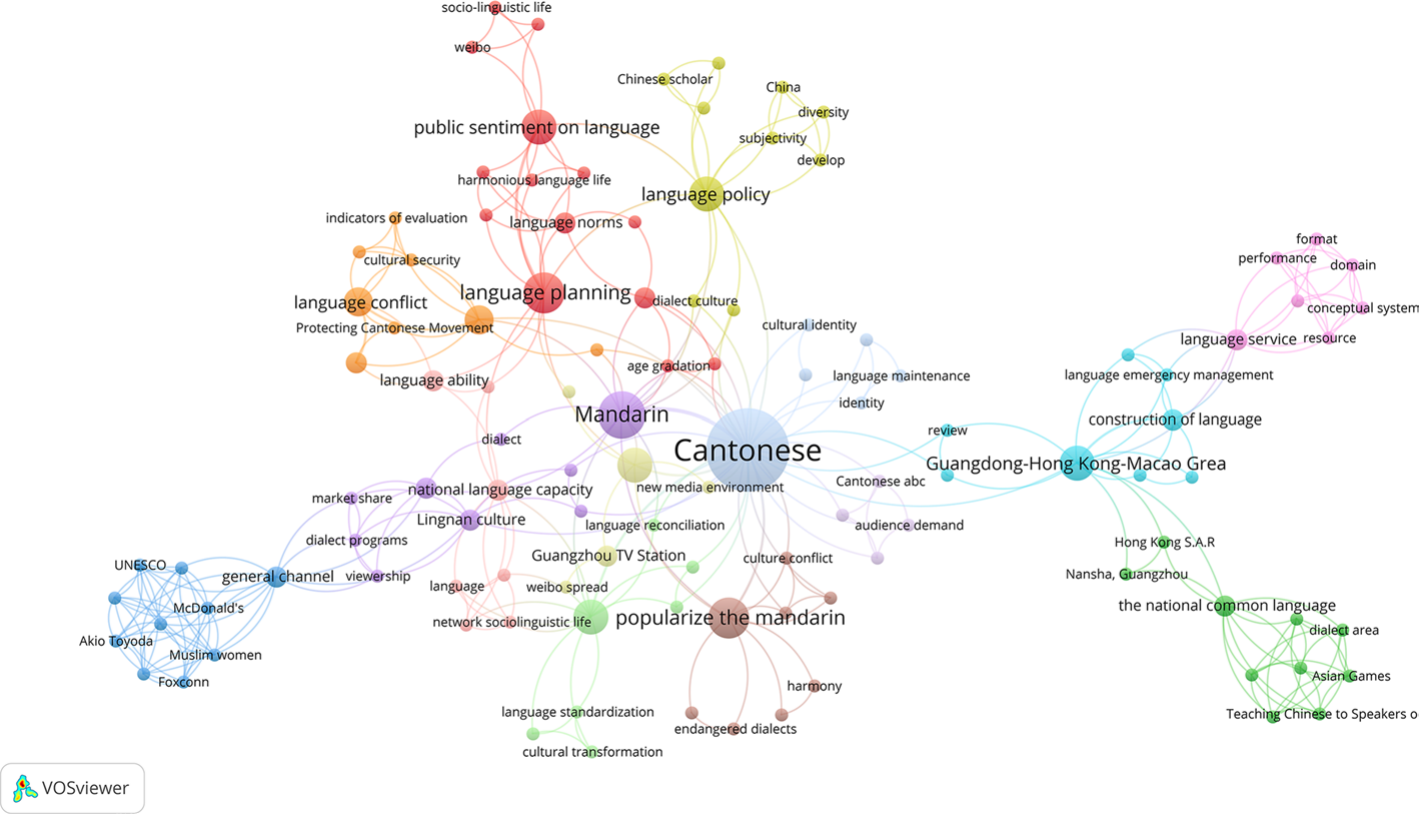
A visual network of keyword co-occurrence in the study of Guangzhou PCM

As shown in Fig. 4, the visual network of keyword co-occurrence was obtained through the keyword co-occurrence analysis of VOSviewer. It showed that the larger the circle, the higher the frequency of the keywords; the thicker the line connecting the two circles, the closer the relationship between the two keywords. When the minimum number of keyword occurrences was limited to 3, the general keyword “Cantonese” was removed. The results showed that the Chinese literature concentrated on the research of Cantonese preservation, promotion of Mandarin, language planning, language policy, and language conflicts.

**Fig. 5:**
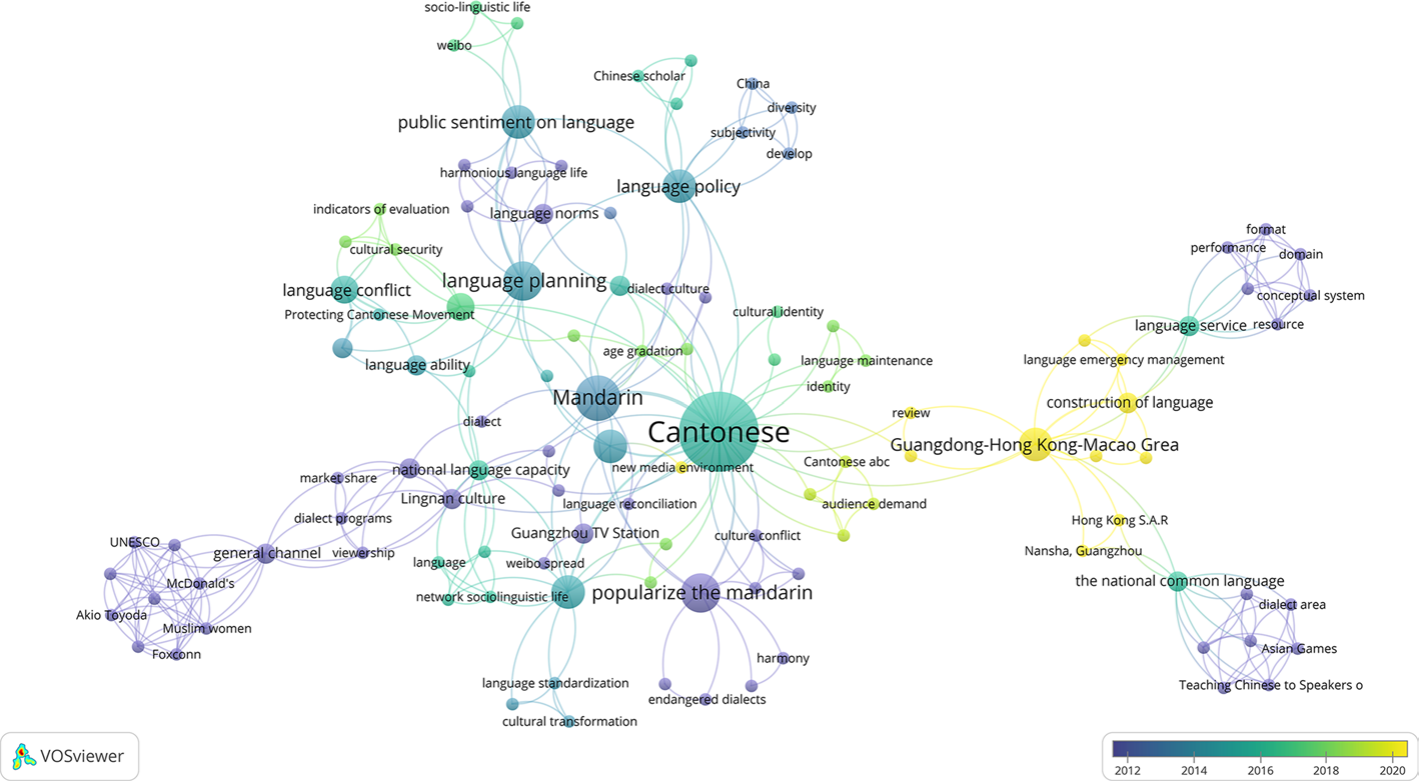
Keyword Coverage Knowledge Graph of Guangzhou PCM

Figure 5 shows the evolution of keywords. During 2010–2013, the research highlighted the promotion of Mandarin, Lingnan culture, language norms, resources, harmonious society and internet dissemination. During 2014–2016, the focalized research was on Mandarin, language planning, language policy and culture, while language conflict was the focus of 2014–2018. Beginning in 2016, the research focus became broader, including Cantonese dialect, language conflicts, national language competence, language service, and language life; from 2019 to 2021, the focus shifted to language construction in the Guangdong-Hong Kong-Macao Greater Bay Area (Greater Bay Area, GBA). The evolution of keywords reflected researchers’ rational thinking on “Protecting Cantonese while promoting Mandarin” and broad thinking on its relevant issues.

## Focus of PCM research

Among the PCM research topics above, the focus of the literature is on the interpretation of the movement, its causes, countermeasures and suggestions.

### Interpretation of PCM

Ten articles, including one English journal paper published between 2010 and 2013, described and analyzed the debate between “promoting mandarin” and “protecting Cantonese” (Chen, 2011; Qu, 2011a, 2011b; Xiao, 2010; Zhan, 2011) in this movement, with more emphasis on public reaction and official reactions.

In nature, PCM started on June 6, 2010 as a “pseudo proposition” via Sina Weibo (microblog), one of the most popular social media platforms in China, and finally shifted into a “true event” in late July due to extensive attention and even participation from all walks of life and official declares (Qu, 2011a, 2011b; Zhan, 2011). Su Zhijia, the former deputy secretary of the Guangzhou Municipal Communist Party Committee (GMPC), publicly stated, “There is no such thing as ‘Mandarin pushing out Cantonese’. Instead, the two varieties of language are complementary to each other” (*Southern Daily*, 2010). To convince the public, on July 28, 2010, the Guangzhou Municipal Government declared at a press conference that the pseudo proposition and the movement should be stopped to avoid public turmoil. This view was further confirmed on August 4th by Mr. Wang Yang, the former secretary of the Guangdong provincial Communist Party Committee, who reiterated that PCM was a nonsense farce. As a result, this movement, which involved an online discussion, a series of spontaneous demonstrations and official reactions, ended after approximately two months.

From the perspective of ideological discourses, Gao (2012) interpreted this event as Chinese netizens’ recontextualization of the political establishment’s official discourses to pursue their linguistic rights at a time when Chinese society has become increasingly pluralistic. To them, Cantonese is not a dialect but a regional lingua franca bonded by social, cultural and linguistic ties (Yan, 2010).

### Causes of PCM

Language policy decisions are significantly mediated by various contextual conditions, both linguistic and non-linguistic, which include sociolinguistic, sociocultural, socioeconomic, and socio-political contexts (Spolsky, 2004). Through the analysis of the above literature, it is found that there are many reasons for preserving Cantonese, which can be categorized into the following four factors.

#### Sociocultural context

The proposed switch from broadcasting in Cantonese to Mandarin by CCPPCC was intentionally to improve the sociocultural environment (“soft” environment in Gao’s words). However, it caused uproars among local Cantonese-speaking residents, especially netizens in Guangzhou (Gao, 2012).

Most of the authors revealed the profound social and cultural factors behind the PCM, among which the relationship between dialect and identity received heated discussion. PCM appeared to be a language dispute on the surface but implied cultural and social conflicts, the confrontation between regional culture and alien culture, and the struggle between cultural diversity and cultural integration in Guangzhou (Zhou, 2013). It reflects people’s “nostalgia” sentiment, which is a social phenomenon and more a cultural phenomenon (Li, 2017). PCM reflected the government’s insufficient attention to protecting Cantonese as a recognition and identity of the Cantonese group (Han, 2013). Thus, PCM is Cantonese speakers’ instinctive response to the increasing impacts and threats to their dialect (Feng, 2011). Cantonese culture should be respected and promoted based on a better understanding of its relationship with Mandarin. After all, Mandarin is not the opposite of Cantonese. (Xiao, 2010).

#### Sociolinguistic context

Language is central to social interaction in every society, regardless of location and time.Some authors (Cai, 2016; He, 2017; Wang, 2015; Zhan, 2011) believe that the occurrence of PCM is due to the change in the social and linguistic environment in which it is located.

First, the sociolinguistic environment failed to popularize language knowledge and language policy to local people. Most of the people who were involved in the “protecting Cantonese” turmoil had special feelings of love for their own dialect and its culture, but they had little knowledge about the relationship between language and dialect. Therefore, a lack of rational understanding of the cause of this incident resulted in the so-called language conflicts (Zhan, 2011).

Second, people have difficulty adapting to the changing sociolinguistic environment. The advancement of urbanization in Guangzhou strengthens the bilingual environment; mandarin as a communicative tool for life and business inevitably compresses the living space of dialects, making local people panic (Cai, 2016; He, 2017). Hence, PCM is not just a simple social and cultural movement but the competition for language use space among different stakeholders, accompanied by conflicts of practical interests (Wang, 2015).

#### Socioeconomic context

The social language environment changes with the development of the local economy. Cantonese was assigned the role of the carrier of cultural tradition, and the rise or fall of a particular regional language depended on regional socioeconomic developments. (Gao, 2017).

To achieve common interests, it is necessary to promote Mandarin in the development of the market economy. However, this normal phenomenon triggered the sensitive nerves of some people, who began to protect dialects with slogans, regarding Mandarin as an imaginary enemy (Liu, 2013). With a large population, the movement reflected the trend that China’s language situation is changing from a basically homogeneous, single, static rural state to a heterogeneous, pluralistic, and dynamic urban state, resulting in language competition, variation, diversion, and fusion (Cai, 2016). Therefore, the language choice for local TV stations to broadcast is a purely commercial act of audience choice out of interest, regardless of dialect protection (Huang, 2010).

#### Media environment

The media texts maintained the national policy of promoting Putonghua (Mandarin) as inviolable (Gao, 2017) and increased people’s awareness of language preservation during and following the PCM.

Weibo, a leading social media platform for people to create, share and discover content online in China, triggered public attention to PCM (Wang, 2010) through a series of incidents: information misreading, celebrity fermentation and group seduction. It soon gathered more netizens or fans, who produced more irrational opinions and depressed emotions for further diffusion (Zhang & Wu, 2017).

Street demonstrations in PCM reflected ordinary citizens’ distrust of government and their desire for more linguistic rights to know, participate and voice their opinions on language policy (Zhang & Wei, 2011). Therefore, they appropriate their actions as powerful weapons in defense of their linguistic rights (Gao, 2012).

PCM is also considered an adjustment of the media language, which reflects the evolution of the language ecological environment of Guangzhou TV media, the environmental changes in population, social language, culture and media, and the competition in audiences, media and cultures (Shu, 2018).

### Countermeasures and suggestions following the PCM

Researchers have also proposed some countermeasures and suggestions at the political, ideological and sociolinguistic environment levels.

#### At the political level

The government’s reaction to PCM is swift and explicit. With a timely intervention from Guangzhou municipal to Guangdong provincial CPC Committee, the movement ended within two months at a particular time when Guangzhou was preparing the Asian Games (Qu, 2011a, 2011b). To avoid similar incidents in the future, the government should draw a lesson from PCM to make timely and appropriate reactions. Meanwhile, language policy is essential to guide language practice, since language use is both an individual and national behavior (Zhang & Liu, 2012). When promoting Mandarin nationwide, it is also important to set language policies to protect dialects or regional lingual franca, including Cantonese (Li, 2012).

PCM reflected the significance of popularizing national language policies. As this disturbance arose with regional misunderstanding of national language policy, it was urgent to publicize and popularize the Law of the People’s Republic of China on the Standard Language and Characters for appropriate language use of Mandarin and Cantonese (Qu, 2011a; Zhan, 2011). From the perspective of China’s national conditions and the status quo of Putonghua and dialects, it is necessary to adjust relevant language policies and strategies, such as the policy shift from “promoting Putonghua” to “promoting Putonghua and inheriting dialects” (Chen, 2011). It should be noted that the so-called protection of dialects is not equivalent to the protection of cultural relics. Different from the outdated antiques in museums, dialects have practical value and can be protected only through actual inheritance of the language.

The PCM increased the necessity of making regional language policy to meet different needs. In essence, the language conflicts in PCM resulted in the readjustment of language interests of all parties. All sectors of society reacted in a timely manner, reenacting laws and policies suitable for the local context (Wang, 2015). Liang (2020) further proposed the compilation of an official Cantonese orthodontic dictionary.

#### At the ideological level

Ideology is the direct factor that determines the occurrence of behavior, which causes Cantonese speakers to protect Cantonese and reject Mandarin in their mind (Shi, 2015; Zhao, 2015). The inappropriate ideology in the Cantonese-speaking region would lead to language conflicts and language education problems. Therefore, people’s updated language perceptions come before their actions to ensure the positive effect of protecting a dialect (Shi, 2015; Zhao, 2015). Many senior Cantonese expressed their ideas such as “I **don’t want** to learn Mandarin” and “I **don’t like** to speak Mandarin” (Wu, 2018).

It is hardly surprising that Cantonese-speaking residents in Guangzhou have closely associated the use of Cantonese with their regional culture and identities (Gao, 2012). They propagated a fear that the city would no longer be theirs, the threat to limit the use of Cantonese in the public sphere being a clear sign of this (Gao, 2012).

Another major reason is the difficulties for people to adapt to the changes in social and linguistic environments. According to Wu (2018), Cantonese speakers have to overcome two obstacles: psychological obstacles and learning obstacles, with the former representing their ideology of Cantonese and the latter stating the challenges to learning Mandarin.

It is necessary to examine language problems from social development, observing the traditional Chinese philosophy of “harmony in diversity”. In this regard, narrow localism that protects one culture with the sacrifice of another should be restrained (Wang & Liu, 2013, 2013). PCM inspired the institutions to be open and inclusive, allowing people to make their choices on what language to use instead of mechanical intervention (Chen, 2011).

#### At the sociolinguistic environment level

Verbal communication and the language environment are always in a relationship of life and death going hand in hand. The survival of a language depends on the needs of the language users and the sociolinguistic environment (Sun, 2004). He (2017) believes that the deep-rooted cause of this “language conflicts” like PCM is the change of the social and ecological environment of the regional dialects: migrants are constantly pouring into Guangzhou, contributing to the modernization of the city while damaging the vital interests of the local people in many ways, thus causing problems of interpersonal and social identity. To avoid similar situations from happening again in the future, the most important thing is to cautiously protect the social and ecological environment of the regional dialect areas. It is essential to acknowledge how named language, such as Cantonese, is important for identity and social purposes (Li, 2022). Zhang and Chen (2012) believe that in addition to the legal basis and language identity, a good language ecological environment is required to build a harmonious language life in the process of urbanization. Without a good language ecology, it is difficult to build a harmonious language life.

Language harmony pursues the coexistence and co-prosperity of multiple languages and dialects (Zhou, 2005). Contemporary China is essentially a bilingual society where Mandarin and dialects coexist. People use dialects and find emotional sustenance, confidence, and self-esteem in them. The promotion and sustainment of particular languages, like dialects, require concerted efforts at different levels, such as individuals’ motivation to learn and use dialects, as well as favorable local and national language policies (Shen et al., 2020).

## Conclusion

The bibliometric analysis of the retrieved literature shows that researchers’ attention is closely connected with hot spots of society. During the past decade, particularly in the early 2010s, PCM attracted researchers’ attention to preserving Cantonese. When the movement came to an end after a short period, the research diversified and rationalized. Nevertheless, it proposed an increasing concern about language maintenance and language balance to create harmonious language ecology. Different from other Chinese regions, multilanguage use is part of local people’s lives for communication in business, study and life in the Greater Bay Area. Although Mandarin is advised as the first service tool for people to communicate, it is important for people to develop multilingual abilities in this multicultural area (Qu, 2020). Such issues as language use, language service, language emergency management and language informatization should be considered by authorities and policymakers in the construction of the Greater Bay Area (Li & Wang, 2020). Against this background, it would be possible to inherit and harmonize Cantonese language and culture for better bonds with Cantonese-speaking communities worldwide to serve Greater Bay Area development.

In response to the increasing demands in the fight against COVID-19, a language service team, the National Language Services Corps of China, was established in Beijing on April 28, 2022 (MOE P.R.China, 2022). Supported by the Ministry of Education and National Language Council, it was jointly launched by 29 partners. Its mission is to deal with various public emergencies and language barriers, providing services in the national majority language, minority languages, Chinese dialects, sign language, Braille, foreign languages, etc. Future research should focus more on the above language issues.

## Data Availability

The original data of this study are presented as they are. It is the only and main paper where such data are used.
